# Kinesiophobia in individuals participating in trekking and hiking activities: a cross-sectional study

**DOI:** 10.3389/fpsyg.2026.1749713

**Published:** 2026-03-20

**Authors:** Taner Yılmaz, Halil Tanır

**Affiliations:** 1 Department of Physical Education and Sport, Faculty of Sports Sciences, Uşak University, Uşak, Türkiye; 2 Department of Coaching Education, Faculty of Sports Sciences, Uşak University, Uşak, Türkiye

**Keywords:** cognitive-behavioral interventions, fear of movement, hiking, Kinesiophobia, trekking

## Abstract

**Introduction and objective:**

Kinesiophobia stands out as a significant psychological barrier that limits performance and participation in nature-based physical activities. To form the basis for the development of appropriate preventive strategies, this study aimed to determine the levels of kinesiophobia among individuals participating in trekking and hiking activities and to examine their associations with demographic and behavioral factors.

**Methods:**

The Tampa Scale of Kinesiophobia was applied to 518 individuals who participated in trekking and hiking activities on the world-famous Lycian Way on the southern coast of Türkiye.

**Results:**

Higher kinesiophobia scores were observed among females, older participants, and individuals with prior negative experiences, with small-to-moderate effect sizes (η^2^ = 0.011–0.069). A combined smoking and alcohol use variable was modestly associated with higher kinesiophobia scores (η^2^ = 0.021), whereas no statistically significant association was found with income status.

**Conclusion:**

The study highlights the importance of psychoeducational programs, cognitive-behavioral interventions, and confidence-based physical awareness exercises in relation to kinesiophobia among participants in trekking and hiking activities. However, due to the cross-sectional design, the findings should be interpreted as associations rather than causal relationships. Future longitudinal and experimental studies can examine the development of kinesiophobia in different age and experience groups in greater detail. In addition, the investigation of psychophysiological indicators and psychological adaptation processes after injury will make valuable contributions to the literature in terms of the prevention and management of kinesiophobia in trekking and hiking activities.

## 1 Introduction

Trekking and hiking are light-paced walks to reach one point from another in groups. While trekking can last several days or weeks, hiking is usually a shorter walk. These activities allow participants to have a pleasant time while also offering extraordinary experiences. In addition to challenging descents and climbs, rugged terrain conditions, weather changes, long distances, and stages requiring physical endurance can make these events challenging (Prószyńska-Bordas and Baranowska, 2021).

Despite this, trekking and hiking have many positive effects on the participants. Trekking and hiking activities contribute to the strengthening of the bond with nature, increase self-confidence (Hanna et al., 2019), and experiences in nature support social development (Foo, 2016). In addition, reduction of stress, strengthening of the immune system, weight control, an increase in general well-being (Mitten et al., 2016), and prevention of diseases such as hypertension, diabetes, cholesterol, and fatty liver (Lone, 2022) are among the known benefits of trekking and hiking activities.

However, despite all these positive effects, trekking and hiking can lead to the development of various psychological barriers in some individuals. Recent international studies emphasize that kinesiophobia is not limited to clinical populations but also affects physically active and recreationally active individuals, leading to avoidance behaviors and reduced participation in nature-based physical activities (Hamer et al., 2021). In particular, the harsh and rugged conditions of the terrain where these activities are carried out can cause kinesiophobia, which is defined as “a state of abnormal and excessive fear of moving or performing physical activity.” Kinesiophobia is likely to develop in those who experience injuries such as falls, sprains, or fractures during trekking and hiking, those with low physical condition, and individuals who observe another injured participant, even if they are not injured themselves. Kinesiophobia can lead to decreased motivation to engage in physical activity, decreased performance, and increased risk of disability (Sahri et al., 2024). Within the framework of the fear-avoidance model, individuals who perceive movement as a potential threat tend to develop maladaptive avoidance behaviors, which may result in long-term physical inactivity even in otherwise healthy and active populations. Therefore, identifying kinesiophobia in individuals participating in trekking and hiking activities is crucial for ensuring participant safety and improving the efficiency of the event. Evidence from recent international research indicates that psychological barriers such as kinesiophobia may have a stronger influence on physical activity participation than pain intensity itself, particularly in older and physically active individuals (Alpalhão et al., 2022). In this regard, adequate preparation for outdoor sports such as trekking and hiking, selecting appropriate equipment, and systematically examining participants’ psychological states through tools such as clinical assessments and standardized questionnaires are effective in mitigating potential risks (Hamer et al., 2021).

At this point, assessing the psychological state of trekking and hiking participants, particularly the risk of kinesiophobia, is important both in terms of preventing potential injuries and encouraging the use of equipment. Individuals with kinesiophobia experience more anxiety during activity and refuse to use equipment. However, the use of equipment is mandatory due to its numerous benefits, primarily safety. For example, trekking poles improve balance during hiking, reduce the load on the lower extremities, and alleviate stress on the musculoskeletal system (Hawke and Jensen, 2020). In addition, Han et al. (2016) reported that poles reduce metabolic load on steep slopes and increase step efficiency. Scientific research demonstrates the importance of equipment usage in terms of enhancing performance and preventing injuries. Therefore, it should be determined whether participants have kinesiophobia, and those with kinesiophobia in particular should be encouraged to use the equipment.

In this regard, identifying kinesiophobia in individuals participating in trekking and hiking activities is important in terms of safety, performance, and participant satisfaction. Detecting kinesiophobia before the event and developing appropriate interventions allows participants to get the maximum benefit from the event and minimize possible risks. For all these reasons, this study aimed to determine the level of kinesiophobia in individuals participating in trekking and hiking activities and to examine its associations with demographic and behavioral factors. Furthermore, the assessment of kinesiophobia among individuals participating in nature-based physical activities is emerging as a significant requirement not only in terms of individual health and safety but also in terms of planning activities in a more efficient, participant-focused, and sustainable manner. This study makes a novel contribution by examining kinesiophobia in a large sample of recreational trekking and hiking participants within a natural outdoor setting, an understudied population in the existing literature, and by simultaneously considering demographic, experiential, and lifestyle-related factors. The data obtained will serve as a guide for event organizers and professionals in the fields of health and sports science, laying the groundwork for the development of preventive strategies for kinesiophobia. In addition, this study will shed light on further studies that address the relationship between kinesiophobia and nature-based physical activity in a multidimensional way, thereby establishing a theoretical foundation for further studies in related fields.

Accordingly, this study was guided by the following research question: Are kinesiophobia levels among individuals participating in trekking and hiking activities significantly associated with demographic characteristics (age, sex, income) and behavioral factors (previous negative experiences, smoking, and alcohol use)? We hypothesized that female sex, older age, longer participation duration, prior negative experiences, and lifestyle-related risk behaviors would be associated with higher levels of kinesiophobia.

## 2 Materials and methods

### 2.1 Research model

In the study designed to identify kinesiophobia in individuals participating in trekking and hiking activities, the general screening model, one of the quantitative methods, was used.

### 2.2 Participants and data collection procedure

The sample of the study consists of 518 volunteers (*n* = 251 females and *n* = 267 males) participating in trekking and hiking activities on the Lycian Way located within the borders of Antalya and Muğla provinces on the southern coast of Türkiye. Convenience sampling was preferred in the sample selection. Among the reasons why the Lycian Way is preferred are that the route is used extensively by both local and foreign nature sports enthusiasts, it hosts a significant part of the individuals participating in trekking and hiking activities in Türkiye, it offers tracks with different difficulty levels, and, unlike many other tracks, there are various accommodation opportunities along the route. In addition, the Lycian Way’s high appeal in terms of historical and natural riches has broadened the study perspective by increasing participant diversity. A total of 600 Turkish individuals attending the Lycian Way trekking and hiking activities were invited to participate in the study. Among these, 550 individuals agreed to participate and completed the questionnaire. Of these, 32 questionnaires were excluded due to incomplete or missing responses, leaving 518 participants included in the final analysis (response rate: 86.3%). A brief recruitment flow is illustrated below:

Total Turkish individuals attending Lycian Way trekking/hiking activities (*n* = 600)

            ↓

Invited to participate in the study (*n* = 600)

            ↓

Agreed to participate and completed the questionnaire (*n* = 550)

            ↓

Excluded due to incomplete/missing responses (*n* = 32)

            ↓

Included in final analysis (*n* = 518)

The study data were collected using the face-to-face interview method in April, May, and June 2025, following the decision of the Uşak University Social Sciences and Humanities Scientific Research and Publication Ethics Committee dated 07.03.2024 and numbered 2024-57. The purpose of the study was explained to participants before administering the questionnaire, and their consent was obtained. A history of negative experiences related to trekking and hiking activities was assessed using a single-item question asking participants whether they had ever experienced any injuries, pain, or physical difficulties during such activities prior to the current study. Participants responded with a binary yes/no option. While this single-item measure allowed for a concise assessment of past adverse experiences potentially influencing kinesiophobia, it does not capture detailed information regarding the severity, frequency, or recency of such events, which may introduce some ambiguity and potential bias. Single-item measures have been shown to provide acceptable validity in large-scale field studies when comprehensive assessments are not feasible, particularly for capturing clearly defined experiential variables. Nevertheless, this approach may have limited sensitivity in detecting variations in severity or frequency. Although no missing data were observed, and kinesiophobia was assessed using the validated Turkish version of the Tampa Scale of Kinesiophobia (TSK), future studies should incorporate more comprehensive injury-related measures.

### 2.3 Data collection tools

#### 2.3.1 Tampa Scale of Kinesiophobia

The Tampa Scale of Kinesiophobia (TSK) is a 17-item self-report instrument designed to assess fear of movement and re-injury. The scale was originally developed by Miller et al. (1991) and later published by Vlaeyen et al. (1995) with the authors’ permission. The TSK includes items related to injury/re-injury beliefs and fear-avoidance behaviors during physical activity. Responses are recorded on a 4-point Likert scale ranging from 1 (strongly disagree) to 4 (strongly agree). Items 4, 8, 12, and 16 are reverse-scored, and total scores are calculated by summing all item responses, yielding a possible score range of 17–68. Higher scores indicate higher levels of kinesiophobia. The Turkish version of the TSK was adapted and validated by Yılmaz et al. (2011) and has demonstrated satisfactory psychometric properties in Turkish populations. In the present study, all participants completed the Turkish version of the TSK. Internal consistency reliability in the current sample was assessed and found to be good, with a Cronbach’s alpha coefficient of 0.87. Since the sample consisted entirely of Turkish individuals, no translation procedures were required, and comprehension of the scale items was ensured.

#### 2.3.2 Smoking and alcohol use

Smoking and alcohol use were assessed with a single binary self-report item (yes/no) capturing whether participants engaged in either behavior. Due to the data collection format, it was not possible to reliably distinguish between smoking and alcohol use for separate analyses. This combined measure was included as a general lifestyle-related risk behavior potentially associated with kinesiophobia. Among the 518 participants, 235 (45.4%) reported engaging in smoking and/or alcohol use, whereas 283 (54.6%) reported neither behavior.

This combined assessment was used because separate measures were not available; while not ideal, it provides an overall view of lifestyle risk factors that may relate to kinesiophobia.

### 2.4 Data analysis

All statistical analyses were conducted using SPSS version 25.0 (IBM Corp., Armonk, NY, USA). The data analysis process was carried out in three sequential stages:

In the first stage, the dataset was prepared for statistical analysis. This process included checking for data entry errors, examining missing data, performing reverse coding for negatively worded items of the Tampa Scale of Kinesiophobia, and computing total scale scores. No cut-off point was applied for the Tampa Scale of Kinesiophobia, and all analyses were conducted using continuous TSK scores in order to avoid arbitrary categorization and to preserve statistical power.

In the second stage, the assumptions required for the planned statistical analyses were examined. For comparisons involving two groups, the assumptions of the independent samples *t*-test were assessed. Normality was evaluated using the Kolmogorov–Smirnov test due to the sample size being greater than 50, and the results indicated that the normality assumption was met for all groups (*p* > 0.05). Homogeneity of variances was examined using Levene’s test, and variance homogeneity was confirmed across all comparisons (*p* > 0.05).

For comparisons involving three or more groups, the assumptions of one-way analysis of variance (ANOVA) were examined. Normality was again assessed using the Kolmogorov–Smirnov test, and all groups met the normal distribution assumption (*p* > 0.05). Homogeneity of variances was evaluated using Levene’s test, and the assumption was satisfied for all ANOVA comparisons (*p* > 0.05).

In the third stage, inferential statistical analyses were performed. Independent samples *t*-tests were used for comparisons between two groups (gender, smoking–alcohol use, previous participation in the event, and experiencing negativity). One-way ANOVA was used for comparisons involving three or more groups (age group, duration of participation in activities, and income status). The significance level (α) was set at 0.05 for all primary analyses. When a statistically significant result was obtained in one-way ANOVA, Tukey’s Honestly Significant Difference (HSD) test was used for *post hoc* multiple comparisons.

To control the Type I error rate in multiple comparisons, Bonferroni correction was applied. Since all ANOVA comparisons involved three groups, the total number of pairwise comparisons was three, and the adjusted significance level was calculated as α = 0.05/3 = 0.017.

Effect sizes were calculated using eta squared (η^2^) for independent samples *t*-tests, one-way ANOVA, and *post hoc* comparisons where statistically significant differences were observed. Effect size estimates provide information about the practical significance of the findings beyond statistical significance. Eta squared values were calculated using the formula proposed by Kahraman et al. (2021):


η=2t/2(t+2N1+N2-2)

The magnitude of effect sizes was interpreted according to Cohen’s (2013) criteria: small (0.01), medium (0.06), and large (0.14). The results of the independent samples *t*-tests, one-way ANOVA, and *post hoc* multiple comparisons are presented in the section “3 Results.”

## 3 Results

The results of the independent samples *t*-test conducted by gender showed that females had significantly higher kinesiophobia scores than males [*t* = 2.412, *p* = 0.016 and Eta squared (η^2^) = 0.011 (small magnitude)]. When comparing smoking and alcohol use, higher kinesiophobia scores were observed in the user group compared to the non-user group [*t* = 3.332, *p* = 0.001, Eta squared (η^2^) = 0.021 (small-to-moderate magnitude)]. Participants who had previously attended the event scored significantly higher than those who had not [*t* = 6.166, *p* < 0.001, Eta squared (η^2^) = 0.069 (moderate magnitude)]. Among the participants, 235 reported smoking and/or alcohol use, while 283 reported neither. As noted in the section “2 Materials and methods,” these behaviors were combined into a single binary variable. The kinesiophobia scores of participants who had previously experienced negative events during trekking and hiking activities were significantly higher compared to those who reported no such experiences [*t* = 4.942, *p* < 0.001, Eta squared (η^2^) = 0.045 (small-to-moderate magnitude)]. The results of the statistical analyses are presented in Tables 1, 2.

**TABLE 1:** Results of the independent sample *t*-test comparing the Tampa Scale of Kinesiophobia scores according to demographic and psychosocial variables.

Variables	Groups	*n*	$\bar{\text{X}}$	Sd±	S_Error_	*T*-test		Eta squared (η ^2^)
						t	*p*	
Gender	Female	251	48.10	7.80	0.49	2.412	0.016*	0.011
	Male	267	46.02	7.70	0.47			
Smoking and alcohol use	Yes	235	48.30	7.80	0.51	3.332	0.001*	0.021
	No	283	45.70	7.70	0.46			
Prior participation in the event	Yes	397	47.07	7.65	0.35	6.166	0.000*	0.069
	No	121	40.83	6.96	0.87			
Experiencing negativity	Yes	173	48.65	7.41	0.56	4.942	0.000*	0.045
	No	345	45.12	7.79	0.42			

*At 0.05 significance level.

**TABLE 2:** Results of one-way analysis of variance (ANOVA) for comparing Tampa Scale of Kinesiophobia scores according to demographic and psychosocial variables (Bonferroni-corrected).

Variables	Groups		*n*	$\bar{\text{X}}$	Sd±	F	*p*	Difference (α = 0.017)	Eta squared (η ^2^)
Age groups	1	18–34 years old	202	42.75	6.85	5.224	0.001*	1–2 2–3	0.063 0.123
	2	35–49 years old	176	46.40	7.18				
	3	50 years old and older	140	48.05	7.47				
	4	Total	518	45.80	7.43				
Duration of participation in events	1	1–2 years	116	45.50	7.65	4.782	0.006*	1–2	0.037
	2	3–4 years	242	48.30	7.54				
	3	5 years and more	160	49.25	7.92				
	4	Total	518	46.30	7.84				
Income status	1	Income less than expenses	54	45.87	6.86	1.009	0.367		
	2	Income equivalent to expense	346	46.83	7.69				
	3	Income more than expenses	118	46.21	8.72				
	4	Total	518	46.30	7.84				

*At 0.05 significance level.

One-way ANOVA revealed significant differences in kinesiophobia scores between age groups [*F* = 5.224, *p* = 0.001] and duration of participation groups [*F* = 4.782, *p* = 0.006]. Individuals aged 35–49 and 50+ had significantly higher scores than those aged 18–34, with moderate effect sizes (η^2^ = 0.123 and 0.063, respectively), while the difference between 35–49 and 50+ was not significant after Bonferroni correction (η^2^ = 0.013, small). Regarding duration of participation, individuals with 1–2 years of participation had significantly lower scores than those with 3–4 years (η^2^ = 0.037, small-to-moderate) and 5+ years (η^2^ = 0.055, moderate), whereas no significant difference was observed between the 3–4 years and 5+ years groups. No significant differences were found between income status groups [*F* = 1.009, *p* = 0.367].

## 4 Discussion

Musculoskeletal pain is a common health problem that negatively affects the quality of life of individuals and can lead to loss of workforce and functional limitations. Not only biomedical factors but also psychosocial factors play a decisive role in the management of these pains. Kinesiophobia, one of the psychosocial factors, is closely related to pain severity and participation in physical activity in individuals with chronic pain. Kinesiophobia can trigger movement avoidance behaviors due to fear of pain or re-injury, leading to limitations in activities of daily living and loss of physical fitness (Luque-Suarez et al., 2018). Consistent with these findings, recent studies conducted in both clinical and non-clinical populations have demonstrated that higher levels of kinesiophobia are associated with reduced physical activity participation, impaired functional capacity, and lower quality of life (Khanna et al., 2022).

Participants were also asked about prior negative experiences during trekking or hiking activities. In this study, “negative experience” specifically referred to incidents of injury, pain, or physical difficulties encountered during past activities. Participants reported whether they had experienced such events at any time prior to the current study. Although measured with a single item, the question was designed to be clear and understandable for all participants. This approach allowed us to capture relevant past experiences while keeping the survey concise. Single-item measures may provide acceptable validity in large-scale field studies when comprehensive assessments are not feasible, particularly for clearly defined experiential variables. However, this approach may have limited sensitivity in capturing the severity, frequency, and timing of such experiences and may be subject to recall bias. Therefore, future studies should employ multi-item or validated retrospective injury assessment tools to improve measurement precision and reduce potential bias.

In trekking activities, particularly during downhill walking, muscles frequently perform eccentric contractions, meaning they lengthen under tension to control movement. Such contractions are associated with Delayed Onset Muscle Soreness (DOMS), which can appear 24–72 h after activity and may lead to temporary discomfort and reduced confidence in movement (Chen et al., 2018; Hody et al., 2019). During eccentric contractions, muscles absorb energy applied by external loads, which is why this action is often referred to as “negative work” (Lindstedt et al., 2001). Individuals experiencing DOMS may perceive movement as potentially harmful, further reinforcing fear of injury and kinesiophobia. However, appropriate preconditioning and low- to moderate-intensity eccentric exercises can attenuate DOMS severity and allow safe participation (Chen et al., 2018; Hody et al., 2019). Trekking organizers and exercise professionals should anticipate that participants may experience temporary muscle soreness due to eccentric loading during descents, and implement guidance, training, and safety measures to reduce kinesiophobia and promote confident, safe movement.

The association of kinesiophobia on participation in physical activity and quality of life is evaluated together with many biopsychosocial variables. Among these variables, age, gender, history of previous injuries, and level of physical activity stand out (Wlazło et al., 2025). In particular, gender can be related to both pain perception and kinesiophobia. In this study, the kinesiophobia levels of female participants were found to be significantly higher than those of males. This finding is consistent with the literature showing that females perceive pain more intensely and are more sensitive to the fear of re-injury (Wiesenfeld-Hallin, 2005; Shah et al., 2017; Vambheim and Øien, 2017; Shaefer et al., 2018).

Similar gender-related differences have also been reported in recent international studies involving physically active populations, suggesting that females may exhibit higher injury-related fear and kinesiophobia even in recreational sports settings (Doğan and Taşcı, 2022).

It is considered that females’ tendency to be more cautious in risky or physically demanding activities is associated with higher levels of kinesiophobia through increased movement avoidance. Similarly, it is reported that females have higher kinesiophobia scores than males in patients with lumbar spinal stenosis and knee osteoarthritis (Perrot et al., 2018; Apaydın et al., 2024). In addition, some studies show that kinesiophobia is common in females with musculoskeletal pain and is seen at higher levels compared to males (Yavaş Çelik et al., 2018; Bingöl et al., 2025).

Lifestyle and habits can be associated with kinesiophobia. Smoking and alcohol use can negatively affect musculoskeletal health and psychological state, leading to increased kinesiophobia. In this study, it was observed that individuals who smoked and consumed alcohol had higher levels of kinesiophobia. A limitation of the present study is that smoking and alcohol use were assessed using a single item, preventing separate analyses of these behaviors. Future studies should consider evaluating these behaviors separately with more detailed measures. This association can be explained by the fact that these habits negatively affect musculoskeletal functions, increase pain perception, and reduce physical endurance. In addition, through psychological effects, fear of re-injury and increased sensitivity to pain increase movement avoidance behaviors. Similar findings have been reported in studies with individuals with chronic low back pain and knee osteoarthritis (Perrot et al., 2018; Yavaş Çelik et al., 2018; Doğan and Taşcı, 2022; Ateş Numanoğlu et al., 2023; Ýnce Parpucu et al., 2023; Apaydın et al., 2024; Ergün et al., 2024; Bingöl et al., 2025). These findings indicate that lifestyle habits are associated with kinesiophobia.

A history of participation in trekking and hiking activities has also been found to be associated with higher TSK scores. This association may reflect reverse causality or selection effects, as individuals with higher fear of movement might be more cautious or more likely to engage repeatedly in supervised trekking/hiking activities. Therefore, these results should not be interpreted as prior participation causing higher kinesiophobia. Individuals with a high level of physical awareness are also more sensitive to signs of pain or injury, which reinforces this situation. Additionally, individuals who have experienced minor injuries over time may accumulate fear-related beliefs, further strengthening movement-related anxiety. It is also emphasized in the literature that past experiences are decisive on kinesiophobia (Kochai et al., 2019; Doğan et al., 2020; Kvist and Silbernagel, 2021; Ohji et al., 2021; Burke, 2022). Furthermore, participants were asked about negative experiences during trekking and hiking activities through a single-item question, which aimed to capture prior injuries or difficulties that might be associated with fear of movement. All responses were completed without missing data, ensuring clarity in the measurement of this variable. Finally, a limitation of the present study is that all participants were Turkish individuals. Although the Lycian Way attracts both domestic and international tourists, foreign participants were not included. Therefore, the findings may only be generalizable to Turkish trekking and hiking participants. Moreover, the Turkish version of the Tampa Scale of Kinesiophobia (TSK) was administered, and comprehension was ensured for all participants. No translation procedures were necessary, as all respondents were native Turkish speakers. Although significant associations were observed between kinesiophobia and factors such as age, gender, lifestyle habits, and prior negative experiences, these findings should be interpreted with caution. The observed associations between kinesiophobia scores and demographic or psychosocial variables indicate relationships rather than causal effects, as multivariable adjustments were not performed. Potential confounding factors may have influenced these associations. Future studies should consider multivariable analyses to clarify independent effects and control for confounders.

The age of individuals is another significant factor that influences kinesiophobia levels. As age progresses, muscle function, metabolic processes, and physiological responses change; this can lay the groundwork for an increased fear of movement. The literature indicates that kinesiophobia increases with age, and that musculoskeletal problems and psychosocial factors are associated with this increase (Kocjan, 2015; Savcun Demirci et al., 2021; Seyhan et al., 2024). A recent scoping review further highlighted that kinesiophobia may act as a more dominant determinant of physical activity behavior than pain itself in older adults, reinforcing the importance of addressing psychological barriers in nature-based activities such as trekking and hiking (Alpalhão et al., 2022).

On the other hand, a direct correlation was found between the year of participation in activities and kinesiophobia. It was determined that individuals who participate in activities for a longer period of time have increased levels of kineesiophobia. This situation can be explained by the fact that as the duration of experience increases, individuals become more aware of possible injury, pain, or decreased performance, which in turn may be related to movement-related anxiety. Recent studies indicate that increased physical activity does not always reduce kinesiophobia and may be associated with increased anxiety levels, particularly in individuals with a history of recurrent injuries (Doğan and Balbaloğlu, 2022; Saldıran et al., 2022).

It was determined that income level did not have a significant association with kinesiophobia. This finding reveals that fear of movement is mostly associated with psychological, physiological, and experiential variables, and economic factors are not decisive (Karadeniz et al., 2023; Kısa et al., 2023).

## 5 Conclusion

As a result, the main variables associated with kinesiophobia levels of trekking and hiking participants were determined as age, gender, year of participation in activities, lifestyle habits (smoking and alcohol use), and past physical experiences. The fact that the association of income level with kinesiophobia was not found to be significant indicates that fear of movement is more related to psychological and experiential factors. The findings highlight the importance of strategies such as psychoeducational programs, cognitive-behavioral interventions, and confidence-based physical awareness exercises aimed at reducing kinesiophobia in trekking and hiking participants. Future longitudinal and experimental studies can examine the development of kinesiophobia in different age and experience groups in greater detail. Investigating psychophysiological indicators and psychological adaptation processes after injury will make valuable contributions to the literature in terms of the prevention and management of kinesiophobia in trekking and hiking activities.

It is important to emphasize that these associations do not imply causality due to the cross-sectional design and lack of adjustment for potential confounders.

## Data Availability

The raw data supporting the conclusions of this article will be made available by the authors, without undue reservation.
